# Impact of a Finnish reform adding new sickness absence checkpoints on rehabilitation and labor market outcomes: an interrupted time series analysis

**DOI:** 10.5271/sjweh.4122

**Published:** 2023-11-01

**Authors:** Mikko Laaksonen, Jenni Blomgren, Hanna Rinne, Riku Perhoniemi

**Affiliations:** 1 Finnish Centre for Pensions (ETK), Helsinki, Finland.; 2 The Social Insurance Institution of Finland (Kela), Helsinki, Finland.

**Keywords:** employment, vocational rehabilitation, rehabilitative psychotherapy, discretionary rehabilitation, medical rehabilitation, sickness allowance, work disability, intervention, work participation, return to work, quasi-experimental study

## Abstract

**Objectives:**

In 2012, new checkpoints were introduced in the Finnish sickness absence system to improve early detection of long-term work disability and hasten return to work after illness. We examined whether the reform affected participation in rehabilitation and labor market outcomes over a one-year period.

**Methods:**

We used interrupted time series analysis among persons who started receiving sickness allowance up to three years before and up to two years after the reform. Separate analyses were conducted among those who passed 30, 60, and 90 sickness allowance days. Poisson regression analysis was used, controlling for seasonal variation, gender, age, and educational level.

**Results:**

After the reform, participation in rehabilitation within one year of passing 30 sickness allowance days increased by 5.1% [incidence rate ratio (IRR) 1.051, 95% confidence interval (CI) 1.015–1.086]. The increase after 60 and 90 sickness allowance days was slightly larger. Looking at the type of rehabilitation, vocational rehabilitation from the earnings-related pension scheme increased most. Regarding the rehabilitation provided by the Social Insurance Institution of Finland (Kela), vocational rehabilitation, medical rehabilitation, and discretionary rehabilitation increased, but the increase was statistically significant only in the last case. Post-reform changes in employment, unemployment, sickness absence and disability retirement were negligible.

**Conclusions:**

The introduction of new sickness absence checkpoints was associated with an increase in participation in rehabilitation but did not affect labor market outcomes one year later. The reform thus was only partially successful in achieving its objectives. Future research should focus on identifying the most effective approaches for utilizing rehabilitation to enhance labor market participation after sickness absence.

Extending working lives by increasing labor force participation has been set as an important policy objective in most Western countries (1, 2). Each year, a large number of working days is lost due to sickness absence and disability pensions. Reducing the number of working days lost due to sickness and disability is therefore a key means of increasing participation in employment. Studies from various countries show that the sooner a person can return to work, the better the chances that the working career continues (3–5).

With the aim of promoting return to work, a wide range of measures of work reintegration and activation of sick-listed employees have been introduced in different countries over recent decades (6, 7). In Finland, various checkpoints were introduced to the sickness absence system in 2012 to improve the chances of detecting long-term disability and hastening return to work after illness (8). First, after 30 days of sickness absence, the employer has to inform the occupational health services about the absence. This new obligation was important for keeping track of sickness absence days especially in cases where the sickness certificate was received from healthcare providers other than occupational healthcare. Second, another amendment shortened the deadline for applying for sickness allowance (SA) from four to two months, corresponding approximately to 60 days. The purpose was to strengthen the existing legislation, under which the rehabilitation needs of a client had to be assessed at the latest when SA had been paid for 60 days. Third, after 90 days of SA, a new requirement stated that the employee must provide a statement from an occupational health physician on his or her remaining work ability and the possibilities to continue working before the SA can be continued.

So far, few studies have examined the effects of the reform. One study found that among public sector employees, return to work after 60 SA days occurred earlier after the reform but there was no change after 30 or 90 days of sickness absence (9). Another study found that return to work after a continuous period of at least 30 SA days increased and the time to return to work shortened after the reform. Changes after the 60 or the 90 SA days were not examined (10). A third study found no increase in return to work after 60 SA days while sickness absence and full disability pensions reduced and the use of partial disability benefits and rehabilitation increased (11). However, the main limitation in all these studies is that they were based on rather simple comparison of SA periods starting in 2010 and 2013. It remains therefore possible that factors other than the policy reform, such as the underlying trends in employment and rehabilitation, may have influenced the findings.

This study adds to the previous literature by examining the impact of the 2012 policy reform using interrupted time series analysis, which offers better possibilities to separate the effects of the reform while controlling for underlying time trends and potential seasonal variations (12–14). In particular, we examine whether the reform influenced participation in rehabilitation and various labor market outcomes (employment, unemployment, sickness absence, and disability pension) over a one-year follow-up period.

## Methods

We used a dataset including the whole Finnish population aged 16–67 years, compiled from the registers of the Social Insurance Institution of Finland (Kela), the Finnish Centre for Pensions, and Statistics Finland.

The SA reform in 2012 concerned SA periods that started after 1 June 2012. For this study, all persons who started a new SA period up to 3 years before (1 June 2009–31 May 2012) and up to 2 years after the reform (1 June 2012–31 May 2014) were extracted from the national SA records. In Finland, the maximum number of SA days due to the same illness is restricted to 300. Because SA is paid for six days a week, this corresponds to approximately one year in calendar time, after which a temporary or a permanent disability pension can be granted (15). The number of SA days is calculated over a period of two years to allow short breaks between separate SA spells. However, if one’s work ability is restored for at least one year, the calculation of the SA days starts from the beginning. Therefore, those who had received SA or disability pension during the previous 12 months were excluded. After this, those who passed 30, 60 and 90 SA days during the maximum accumulation period of SA were selected. For consistency, we calculated compensated SA days for each of the checkpoints, although the 30-day rule refers to calendar rather than compensation days, and the 60-day criterion is a combination of the two.

### Outcome variables

Participation in rehabilitation was examined cumulatively between the beginning of the SA period and one year after passing the 30, 60 or 90 SA days mark.

The dataset included rehabilitation organized by Kela and the pension insurers. Kela organizes vocational rehabilitation, intensive medical rehabilitation, and rehabilitative psychotherapy as well as other vocational or medical rehabilitation as discretionary rehabilitation (16). Discretionary rehabilitation is complementary to other forms of rehabilitation and is allocated based on an annual budget approved by the Finnish Parliament. Rehabilitative psychotherapy was separated from discretionary rehabilitation in 2011, that is, during the pre-reform period of this study. We therefore extracted rehabilitative psychotherapy from discretionary rehabilitation also prior to this change, keeping only other forms of rehabilitation in the discretionary rehabilitation category. While Kela’s vocational rehabilitation is intended for the unemployed, students and persons with a short work history, pension insurers organize vocational rehabilitation for those who have established working careers and are either currently employed or have been employed quite recently (17).

Labor market outcomes were measured exactly one year after passing each of the checkpoints. The examined labor market outcomes were employed (and not simultaneously receiving unemployment benefits, SA or disability pension), unemployed, SA and disability pension. Those who were not employed and received none of the benefits were classified in the group “other”.

In practice, the 30- and 90-day checkpoints concern only those who are employed. We therefore also separated those who were and were not employed at the beginning of the SA period. Furthermore, we controlled for gender, age, and the level of education, as these factors are known to be important determinants of sickness absence, rehabilitation and employment outcomes.

### Statistical methods

We first present descriptive information on demographic characteristics and the prevalence of rehabilitation and labor market outcomes in the pre- and post-reform groups. The pre-reform group consisted of those who started their SA period in the three years prior to the reform to better distinguish underlying temporal trends and seasonal patterns (18), while for the post-reform group, two years was considered sufficient. It should be noted that any differences in the background characteristics between these two broad groups do not necessarily imply changes due to the reform, but may also reflect underlying long-term trends, such as gradual improvement of educational level over time.

Interrupted time series analysis applying Poisson regression was used as the main analytical method (12–14). SA periods starting up to three years before the reform and up to two years after the reform were converted into a monthly time series of 60 data points (36 months before the reform and 24 months after the reform). In the interrupted time series analysis, the observed post-reform development is compared to the counterfactual situation of what would have happened without the reform, achieved by extrapolating the pre-reform trend into the post-reform period. Any deviance of the outcome from the counterfactual scenario in the post-intervention period is then attributed to the impact of the intervention. The effect of the reform was modelled by including an indicator variable taking the value 0 before the reform and 1 after the reform. If there was a change in rehabilitation or the labor market outcomes due to the reform, we expect to observe an immediate change at the time of the reform. Seasonality was adjusted for by including Fourier terms (sine and cosine functions) if they were statistically significant at the 0.10 level (18, 19). In addition, the analyses were adjusted for gender, age, and the level of education and stratified by employment status at the beginning of the SA period.

The effects of the reform are presented as incidence rate ratios (IRR), and 95% confidence intervals were calculated from the robust standard errors. Autocorrelation was checked by plotting the autocorrelation and partial autocorrelation functions and was found to be negligible. The analyses were conducted using Stata 16.1, Stata Corp, College Station, TX, USA.

## Results

Around 55% of those who passed 30 SA days were women, while among those who passed 60 or 90 SA days, the proportion of women was slightly lower (table 1). Concerning all SA lengths, the proportion of women was higher in the post- than pre-reform period. Those who passed 60 and 90 SA days were on average slightly older than those passing 30 days. In the post-reform period, the share of the youngest and the highest age groups slightly increased. Those with a higher number of SA days were less highly educated than those with shorter absences. The level of education was slightly higher in the post- than pre-reform period. The proportion of those who were employed when the SA period started was somewhat smaller among those having longer absences, with no marked differences between the pre-reform and post-reform periods.

**Table 1:** Demographic characteristics and study outcomes in the 3-year pre-reform (1 June 2009–31 May 2012) and 2-year post-reform (1 June 2012–31 May 2014) periods among those who passed 30, 60 and 90 sickness allowance (SA) days. Rehabilitation was measured between the start of the sickness allowance period and one year after passing 30, 60 or 90 SA days and labour market outcomes were measured exactly one year after passing 30, 60 and 90 SA days.

			30 SA days			60 SA days			90 SA days	
			Pre-reform (N=305 500)	Post-reform (N=193 663)		Pre-reform (N=194 977)	Post-reform (N=123 000)		Pre-reform (N=144 932)	Post-reform (N=90 002)
			%	%		%	%		%	%
**Background characteristics**										
	Women		54.8	55.3		53.1	53.7		52.5	53.2
	Age group (years)									
		18–24	7.4	8.2		7.3	8.2		7.2	8.3
		25–34	16.4	16.5		14.3	14.7		13.3	13.7
		35–44	19.5	18.9		18.3	17.6		17.9	17.2
		45–54	29.9	28.9		30.7	29.5		31.0	29.9
		55–67	26.9	27.5		29.4	30.0		30.6	31.0
	Level of education									
		Primary	23.3	21.2		25.9	23.6		27.6	25.2
		Secondary	51.0	52.2		51.6	52.8		51.7	53.0
		Tertiary	25.6	26.5		22.6	23.6		20.8	21.8
		Employed at baseline	78.5	78.3		73.4	73.2		69.9	68.9
**Rehabilitation outcomes**										
	Any rehabilitation		10.2	11.6		14.3	16.3		17.8	20.4
	Kela’s rehabilitation		7.9	9.1		10.8	12.3		13.3	15.1
		Vocational rehabilitation	1.6	1.8		2.4	2.7		3.2	3.6
		Medical rehabilitation	0.3	0.3		0.4	0.5		0.6	0.7
		Discretionary rehabilitation	4.3	4.7		5.9	6.3		7.2	7.9
		Rehabilitative psychotherapy	2.0	2.6		2.6	3.4		3.1	3.9
	Vocational earnings-related rehabilitation		1.9	2.5		3.3	4.2		4.6	5.9
**Labor market outcomes**										
	Employed		56.7	54.9		45.0	43.6		35.7	34.0
	Unemployed		11.9	13.9		13.8	16.2		15.3	17.8
	Sickness allowance		6.1	6.3		5.6	5.9		4.5	4.9
	Disability pension		15.8	14.9		25.3	23.6		33.9	32.0
	Other		9.5	10.0		10.3	10.7		10.6	11.3

Table 1 also shows the prevalence of the rehabilitation and labor market outcomes. Within one year of passing 30 SA days, around 10% of the study population had participated in rehabilitation, while among those who passed 90 SA days, the proportion was around 20%. Participation in rehabilitation was slightly more common in the post- versus pre-reform period. Of the rehabilitation provided by Kela, discretionary rehabilitation was most common, followed by rehabilitative psychotherapy. Medical rehabilitation was rare. Participation in the earnings-related vocational rehabilitation was more common than participation in Kela's vocational rehabilitation.

Being employed one year later was less frequent after passing a higher number of SA days, while unemployment and disability pension became more frequent (table 1). Differences between the pre- and post-reform groups were small. However, the outcomes typically seemed less favorable in the post-reform groups, rather than showing improvement.

**Figure 1:**
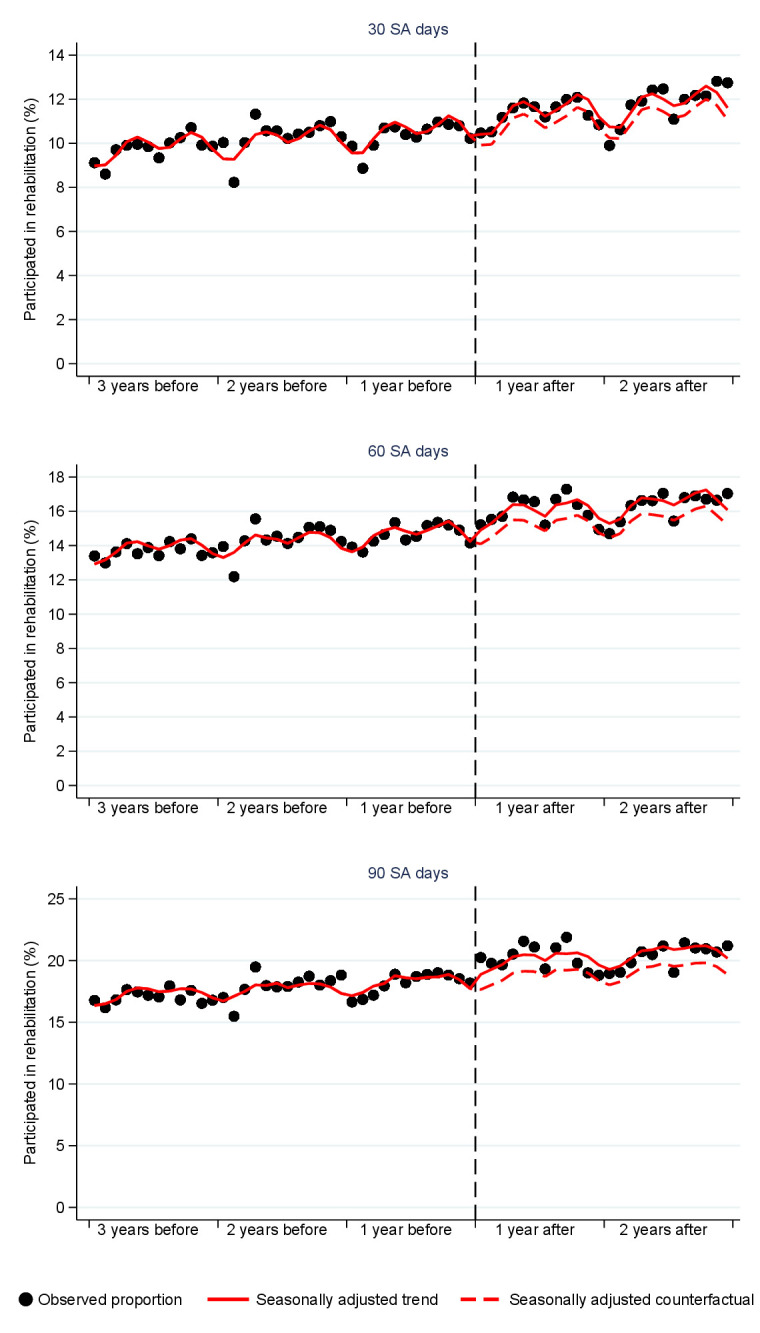
The proportion of those who participated in rehabilitation between the start of the sickness allowance period and one year after passing 30, 60 and 90 sickness allowance (SA) days (%) among those who started their sickness allowance period up to three years before the reform and up to two years after the reform. The circles represent observed monthly prevalences. The solid line is the predicted trend adjusted for seasonality and the demographic confounders and the dashed line is the corresponding counterfactual. The reform has been marked with the dashed vertical line.

Figure 1 presents the proportion of those who participated in rehabilitation within one year after they had passed 30, 60 or 90 SA days among persons starting their SA period three years before and two years after the reform. The circles show the observed proportions of those who participated in rehabilitation, each circle representing the month in which the SA period first started. Over time, the proportion of persons participating in rehabilitation increased. There was also clear seasonal variation with two cycles per year. The solid line shows the seasonally adjusted predicted trend based on Poisson regression model, and the dashed line presents the counterfactual situation in the post-reform period if there had been no reform. Comparing the seasonally adjusted trend with the counterfactual shows that the proportion of people participating in rehabilitation increased in the post-reform period compared to the previous trend, and the increase appeared to be larger for the longer absence durations.

Table 2 presents the IRR for the impact of the reform on the probability of participating in rehabilitation within one year of each checkpoint by the type of rehabilitation. According to the models, the probability of participating in any rehabilitation after a SA period lasting ≥30 days increased by 5.1% (IRR 1.051, 95% CI 1.015–1.086) after the reform. After passing the 60 and 90 SA days, the increase was even slightly larger. Rehabilitation provided by Kela increased by around 4–6%, depending on the checkpoint. However, when examined by the type of rehabilitation, only Kela’s discretionary rehabilitation showed a statistically significant increase. This is affected by the low volumes of Kela’s vocational rehabilitation and medical rehabilitation, which reduces the statistical power of the analyses. The point estimates for these rehabilitation types were of the same order of magnitude as for discretionary rehabilitation. Vocational rehabilitation from the earnings-related pension scheme increased by 8–10%. Overall, changes in rehabilitation after the reform tended to be slightly larger for longer sickness absences.

**Table 2:** Impact of the sickness allowance reform on participation in rehabilitation at one year after passing the 30, 60 and 90 sickness allowance (SA) days, interrupted time series analysis adjusted for seasonality and the demographic confounders, incidence rate ratios (IRR) and 95% confidence intervals (95% CI) from Poisson regression analysis.

			30 SA days			60 SA days			90 SA days	
			IRR (95% CI)	P–value		IRR (95% CI)	P–value		IRR (95% CI)	P–value
**All**										
	Any rehabilitation		1.051 (1.015–1.086)	0.004		1.058 (1.023–1.093)	0.001		1.070 (1.035–1.107)	<0.001
	Kela’s rehabilitation		1.040 (1.002–1.080)	0.04		1.049 (1.009–1.090)	0.02		1.057 (1.016–1.100)	0.006
		Vocational rehabilitation	1.057 (0.974–1.147)	0.18		1.074 (0.991–1.164)	0.08		1.081 (0.998–1.172)	0.06
		Medical rehabilitation	1.097 (0.899–1.339)	0.36		1.096 (0.893–1.346)	0.38		1.146 (0.937–1.402)	0.19
		Discretionary rehabilitation	1.057 (1.007–1.110)	0.03		1.056 (1.001–1.113)	0.04		1.057 (1.003–1.114)	0.04
		Rehabilitative psychotherapy	0.978 (0.912–1.049)	0.54		1.003 (0.927–1.085)	0.94		1.020 (0.936–1.111)	0.66
	Vocational earnings–related rehabilitation		1.086 (1.004–1.175)	0.04		1.090 (1.013–1.172)	0.02		1.095 (1.020–1.175)	0.01
**Employed at the beginning of the SA period**										
	Any rehabilitation		1.044 (1.005–1.084)	0.02		1.053 (1.014–1.094)	0.008		1.074 (1.034–1.116)	<0.001
	Kela’s rehabilitation		1.031 (0.989–1.076)	0.15		1.027 (0.983–1.073)	0.23		1.049 (1.001–1.100)	0.06
		Vocational rehabilitation	1.093 (0.970–1.233)	0.14		1.142 (1.004–1.300)	0.04		1.192 (1.042–1.363)	0.01
		Medical rehabilitation	1.011 (0.783–1.304)	0.94		1.012 (0.778–1.317)	0.93		0.954 (0.726–1.254)	0.74
		Discretionary rehabilitation	1.030 (0.978–1.085)	0.25		1.021 (0.964–1.082)	0.47		1.036 (0.976–1.100)	0.25
		Rehabilitative psychotherapy	0.982 (0.904–1.068)	0.67		0.966 (0.883–1.058)	0.46		1.035 (0.933–1.148)	0.52
	Vocational earnings–related rehabilitation		1.103 (1.018–1.196)	0.02		1.097 (1.018–1.181)	0.02		1.100 (1.024–1.181)	0.01
**Not employed at the beginning of the SA period**										
	Any rehabilitation		1.057 (0.995–1.123)	0.07		1.070 (1.008–1.135)	0.03		1.046 (0.986–1.110)	0.13
	Kela’s rehabilitation		1.069 (1.001–1.143)	0.05		1.084 (1.017–1.156)	0.01		1.057 (0.991–1.127)	0.09
		Vocational rehabilitation	1.014 (0.915–1.125)	0.79		1.035 (0.934–1.146)	0.51		0.991 (0.900–1.092)	0.86
		Medical rehabilitation	1.300 (0.940–1.798)	0.11		1.329 (0.956–1.846)	0.09		1.431 (1.043–1.964)	0.03
		Discretionary rehabilitation	1.149 (1.029–1.284)	0.01		1.144 (1.030–1.271)	0.01		1.110 (1.000–1.233)	0.05
		Rehabilitative psychotherapy	0.967 (0.847–1.105)	0.62		1.023 (0.895–1.169)	0.74		0.966 (0.841–1.109)	0.62
	Vocational earnings–related rehabilitation		1.055 (0.825–1.349)	0.67		1.077 (0.846–1.371)	0.55		1.073 (0.846–1.362)	0.56

Table 2 also shows the IRR stratified by employment status at the beginning of the SA period. As most sickness absence recipients were employed at baseline (around 70–80%, depending on the checkpoint), for them the results were generally fairly similar to those of the whole study population (table 2). However, the estimates for Kela’s vocational rehabilitation increased and were statistically significant for the 60- and 90-day checkpoints. Among those who were not employed at the beginning of the SA, discretionary rehabilitation increased after the reform. Medical rehabilitation showed large point estimates, but the increase was not statistically significant, except for the 90-day checkpoint.

**Figure 2:**
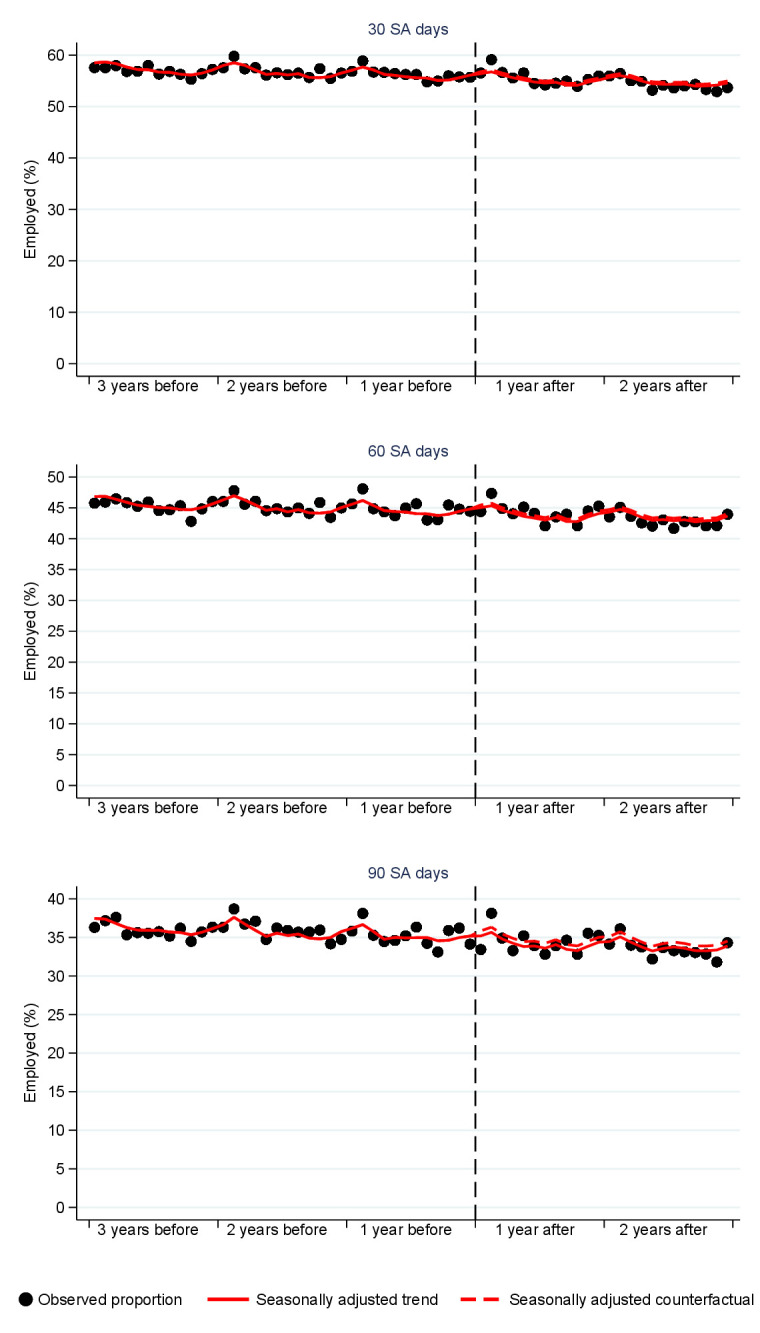
The proportion of those who were employed one year after passing 30, 60 and 90 sickness allowance (SA) days (%) among those who started their sickness allowance period up to three years before the reform and up to two years after the reform. The circles represent observed monthly prevalences. The solid line is the predicted trend adjusted for seasonality and the demographic confounders and the dashed line is the corresponding counterfactual. The reform has been marked with the dashed vertical line.

Figure 2 presents the interrupted time series graph for the probability of being employed one year after passing 30, 60 and 90 SA days. There was a general decreasing trend, and seasonal variation was seen in the form one cycle per year. However, the graph shows very little change in the level of employment due to the reform for any of the sickness absence lengths. This is confirmed in table 3, which shows the modelling results for all labor market outcomes. The changes for other labor market outcomes were also relatively small. However, after the reform, those who passed the 90-day checkpoint were less likely to be unemployed and more likely to receive SA one year later.

**Table 3:** Impact of the sickness allowance reform on labour market outcomes one year after passing the 30, 60 and 90 sickness allowance (SA) days, interrupted time series analysis adjusted for seasonality and the demographic confounders, incidence rate ratios (IRR) and 95% confidence intervals (95% CI) from Poisson regression analysis.

		30 SA days			60 SA days			90 SA days	
		IRR (95% CI)	P-value		IRR (95% CI)	P-value		IRR (95% CI)	P-value
**All**									
	Employed	0.993 (0.982-1.003)	0.16		0.991 (0.975-1.007)	0.25		0.981 (0.960-1.002)	0.08
	Unemployed	0.993 (0.964-1.023)	0.66		0.985 (0.955-1.016)	0.34		0.965 (0.933-0.999)	0.04
	Sickness allowance	1.043 (0.999-1.089)	0.05		1.044 (0.985-1.106)	0.14		1.087 (1.011-1.169)	0.02
	Disability pension	1.001 (0.974-1.029)	0.94		1.009 (0.984-1.036)	0.48		1.008 (0.984-1.033)	0.52
	Other	1.010 (0.978-1.042)	0.55		1.004 (0.966-1.043)	0.85		1.040 (0.997-1.086)	0.07
**Employed at the beginning of the SA period**									
	Employed	0.987 (0.978-0.996)	0.01		0.982 (0.968-0.997)	0.02		0.968 (0.949-0.987)	0.001
	Unemployed	1.042 (0.994-1.092)	0.09		1.026 (0.974-1.081)	0.34		1.017 (0.962-1.076)	0.56
	Sickness allowance	1.026 (0.979-1.075)	0.28		1.049 (0.983-1.119)	0.15		1.077 (1.005-1.153)	0.04
	Disability pension	1.040 (1.007-1.074)	0.02		1.035 (1.003-1.065)	0.05		1.037 (1.002-1.070)	0.03
	Other	0.996 (0.956-1.039)	0.87		0.973 (0.992-1.027)	0.32		1.020 (0.960-1.084)	0.52
**Not employed at the beginning of the SA period**									
	Employed	0.992 (0.931-1.056)	0.80		1.000 (0.925-1.082)	0.99		1.053 (0.956-1.160)	0.29
	Unemployed	0.977 (0.947-1.009)	0.16		0.971 (0.934-1.009)	0.13		0.954 (0.916-0.994)	0.02
	Sickness allowance	1.099 (0.991-1.219)	0.07		0.997 (0.877-1.133)	0.96		1.065 (0.911-1.246)	0.43
	Disability pension	0.967 (0.930-1.005)	0.09		0.980 (0.946-1.016)	0.28		0.993 (0.960-1.027)	0.69
	Other	1.039 (0.994-1.086)	0.09		1.059 (1.006-1.117)	0.03		1.042 (0.981-1.107)	0.18

Among those who were employed in the beginning of the SA period, being employed one year after passing any of the checkpoints became less probable after the reform. Having a disability pension, and for those passing the 90-day checkpoint, continuing on SA became more probable. Among the smaller group of those who were not employed at the beginning of the SA period, statistically significant findings were few. Notably, among those who passed the 90-day checkpoint, unemployment became less probable.

The one-year follow-up time after passing the checkpoints is of course to some extent arbitrary. Results with a two-year follow-up time are presented in the supplementary material (www.sjweh.fi/article/4122, tables S1 and S2). The main findings remain robust.

## Discussion

This study examined whether the reform aiming to improve earlier detection of long-term work disability and hasten return to work after illness affected the probability of participating in rehabilitation and various labor market outcomes over a one-year period. We found that the probability of receiving rehabilitation increased but effects on labor market outcomes were small.

Overall, rehabilitation increased after the reform by 5–7%. The largest increase was seen in vocational rehabilitation from the earnings-related pension system among those who were employed when the SA period started, but the increase was not statistically significant among those outside work. This is understandable, as this rehabilitation is meant for people with relatively stable connections to working life (17).

Of the Kela rehabilitation, vocational and discretionary rehabilitation showed statistically significant increases for at least some of the checkpoints. The estimates for medical rehabilitation were the highest but did not reach statistical significance, likely relating to the low occurrence of this type of rehabilitation in the study population. As a whole, Kela rehabilitation increased more among those who were not at work when the SA period started. However, there was variation between rehabilitation types. Vocational rehabilitation increased after 60 and 90 SA days among those who were at work when the SA period started but there was no increase among those outside work. In contrast, discretionary and medical rehabilitation increased among those not working at baseline but not among those who were at work. Thus, it seems that discretionary and medical rehabilitation have been considered most appropriate to answer to the challenges faced by those outside work while vocational rehabilitation has been directed to the employed.

Psychotherapy is one of the means to increase work participation among those with mental disorders (20, 21). In our study, the reform showed no association with participation in rehabilitative psychotherapy. It may be that referral to rehabilitation for mental health problems is more strongly driven by factors other than external incentives such as passing SA checkpoints. Rehabilitative psychotherapy in Finland requires that the patient has already received appropriate treatment for three months and that it is evaluated that psychotherapy increases work ability or the ability to continue studying. It is the rehabilitee’s own responsibility to find a therapist, and only part of the expenses are covered (22, 23), which may lead to differences in usage between different population groups (24).

The reform had no effect on being in employment one year after reaching any of the sickness absence checkpoints. Among those who were working when the SA period started, employment even slightly decreased. Also, the probability of receiving disability pension slightly increased. One possible explanation for this is that due to increased rehabilitation, intensified examination of work ability has more often revealed valid reasons for granting a disability pension. Also being on SA one year after passing the 90-day checkpoint increased. As receiving SA based on the same illness one year after passing the checkpoint is not possible if the SA period has been continuous, it may be that SA has continued longer than before because there have been breaks caused by rehabilitation.

### Evaluation of the findings

Our findings contradict previous studies that found an increase in work participation after the reform (9, 10). However, the results are more alike with a study published in Finnish, which focused on individuals with ≥60 SA days, and found that return to work slightly decreased after the reform (11). The study was restricted to recently employed persons, making the results more comparable to our findings for those who were employed when the SA started. Also being on sickness absence and disability pensions decreased, while participation in rehabilitation and the use of partial disability benefits clearly increased: over the next 10 months after 60 SA days, the time spent in rehabilitation increased by around 50%. Although the results are not directly comparable due to different measurement units, the increase in rehabilitation was clearly larger than in our study. However, all these previous studies compared SA periods starting in 2010 and 2013, making them susceptible to the underlying trends in employment and rehabilitation. In the aftermath of the Great Recession, the overall employment situation in 2010 was poor but recovered in the subsequent years (25). Participation in rehabilitation also expanded strongly in those years (16, 17). As the study periods were clearly apart, there is also a risk for other changes that may have affected the results.

Overall, in our study, the changes in rehabilitation and labor market outcomes were fairly similar across all checkpoints, although often slightly more pronounced for those with longer absences. Separating the effects of passing the 30-, 60- and 90-day checkpoints is difficult because all changes were implemented simultaneously. All those who passed the 90-day checkpoint had also passed the 30- and 60-day checkpoints a little earlier. Conversely, a large proportion of those who passed the earlier checkpoints also passed the later checkpoints shortly afterwards. Therefore, any measures that resulted from passing one checkpoint may also have affected the findings of passing the other checkpoints.

Across different countries, the procedures for monitoring persons on sick leave show considerable variation. In terms of its demands for the employee and the employer, the Finnish system falls somewhere in the middle, while some countries have higher and others lower requirements (7, 26). The reform in 2012 did not impose specific return-to-work measures but rather mandated regular evaluations to assess the need for timely action. This process can then sometimes lead to initiation of rehabilitation or other measures to support work ability. The results on increased rehabilitation can therefore be considered good. At the same time, the study raises questions about the links between rehabilitation and labor market outcomes. Although we did not specifically examine the association between rehabilitation and employment, the results suggest that increased rehabilitation did not advance employment in the study population. Previous studies on the effectiveness of rehabilitation have provided fairly modest results (27, 28). One possible explanation for this is that rehabilitation is provided quite late in the process of work disability (29). A recent Finnish study found that vocational rehabilitation increased work participation by 7 percentage points compared to a matched control group (30). Another study suggested an effect of similar magnitude (31). In other studies, rehabilitative psychotherapy was found to increase employment up to 6 percentage points in the next five years (23) and reduce work disability by 20% (22). In general, rehabilitation is targeted at people who are expected to benefit most from it. It could be assumed that if the number of people entering rehabilitation increases due to the reform, more individuals with limited employment prospects are included, diluting the overall effect of rehabilitation.

### Methodological considerations

The study was based on comprehensive and reliable register data, including detailed information on SA periods and the outcome measures with exact dates. Interrupted time series analysis offers a strong quasi-experimental study design that can be used to assess the impacts of policy reforms (12–14). It has the advantage of being able to control for pre-existing time-trends and possible seasonal effects, both of which are important features in our study. For example, the change of rehabilitative psychotherapy from discretionary to mandatory in 2011 led to an increase in its use (16), but we have taken this into account in the analysis. Moreover, there is considerable seasonal variation in SA. In particular, SA periods are less likely to start in the summer months and around the New Year’s holiday season, and the characteristics of SA recipients also vary according to the starting date. This seasonal variation is equally taken into account. A limitation in interrupted time series analysis is that it remains possible that other events occurring at the same time with the reform can influence the findings. Controlled interrupted time series analysis (32, 33) would provide an even stronger basis for causal inference, but as the reform was nationwide, such a design was not possible. However, we consider it fairly unlikely that any other major change affecting rehabilitation would have occurred at exactly the same time as the reform.

For simplicity, all checkpoints were measured as compensation days, although this is not exactly how they are formulated in the legislation. The 30-day rule refers to one month of sickness absence, meaning calendar days (including the 10-day employer period), and the so-called '60-day amendment' is strictly speaking defined as two months in calendar time, even though it is also connected to the pre-existing regulation of assessing the need for rehabilitation at 60 SA days. We don’t expect that our decision to define the different accumulation periods systematically as SA days has a significant effect on the results, especially as the effects of passing the various checkpoints are not clearly distinguishable.

The Finnish rehabilitation system includes various providers who offer rehabilitation to different population groups (34). The dataset included rehabilitation organized by Kela and pension insurers but not rehabilitation organized by other bodies, such as medical rehabilitation by the municipalities and physiotherapy by occupational health care. Because it is not always easy to draw a line between treatment and rehabilitation, it is difficult to say exactly what proportion of all rehabilitation is covered. However, the majority of rehabilitation aimed at promoting work ability and supporting people to stay in or return to work is included.

Previous studies have found some subgroup differences in the effects of the reform (9–11), although the differences have been relatively small and not consistent across studies. Future studies should pay attention to the subgroup differences, as they could offer valuable insights into the mechanisms of the reform’s impact.

### Concluding remarks

Our study showed that the introduction of new SA checkpoints increased participation in rehabilitation but did not affect labor market outcomes one year later. Thus, the reform was only partially successful in achieving its objectives. Internationally, there is strong need for finding effective measures to promote return to work and extend working lives. Our study shows that the pathway from rehabilitation to return to work needs to be strengthened. Future research should focus on identifying the most effective approaches for utilizing rehabilitation to enhance labor market participation after different lengths of sickness absence.

### Ethical statement

In Finland, register-based studies do not require an ethical review. However, data collection, analysis and reporting were carried out in accordance with the ethical standards of The Finnish Advisory Board on Research Integrity.

## Supplementary material

Supplementary material
