# Supplementary material for: Impact of a Finnish reform adding new sickness absence checkpoints on rehabilitation and labor market outcomes: an interrupted time series analysis
Source: Scand J Work Environ Health. 2023 Oct 31;49(8):588–97. doi: 10.5271/sjweh.4122 (PMC10881278; doi:10.5271/sjweh.4122)
Supplement: Supplementary material [file SJWEH-49-588-S001.pdf]

Impact of a Finnish reform adding new sickness absence checkpoints on rehabilitation and labor market outcomes: an interrupted time series analysis[1]

By Mikko Laaksonen, PhD,[2] Jenni Blomgren, PhD, Hanna Rinne, PhD, Riku Perhoniemi, MSc

1. Supplementary tables
2. Correspondence to Mikko Laaksonen, Finnish Centre for Pensions, FI-00065 Eläketurvakeskus. [E-mail: mikko.laaksonen@etk.fi]

Table S1. Impact of the sickness allowance reform on participation in rehabilitation at TWO YEARS after passing the 30, 60 and 90 sickness allowance (SA) days, ITS-analysis adjusted for seasonality and the demographic confounders, incidence rate ratios (IRR) and 95% confidence intervals (95% CI)

	30 SA days		60 SA days		90 SA days	
	IRR (95% CI)	p-value	IRR (95% CI)	p-value	IRR (95% CI)	p-value
**All**						
Any rehabilitation	1,047 (1,019-1,076)	0,001	1,055 (1,027-1,085)	<0,001	1,063 (1,033-1,093)	<0,001
Kela's rehabilitation	1,042 (1,009-1,075)	0,01	1,048 (1,014-1,083)	0,006	1,050 (1,014-1,088)	0,006
Vocational rehabilitation	1,086 (1,018-1,160)	0,01	1,084 (1,012-1,161)	0,02	1,090 (1,015-1,170)	0,02
Medical rehabilitation	1,053 (0,890-1,247)	0,54	1,065 (0,895-1,267)	0,47	1,124 (0,945-1,338)	0,18
Discretionary rehabilitation	1,038 (0,996-1,082)	0,08	1,045 (1,000-1,091)	0,05	1,034 (0,989-1,081)	0,13
Rehabilitative psychotherapy	1,013 (0,951-1,079)	0,69	1,022 (0,951-1,099)	0,55	1,028 (0,949-1,114)	0,49
Vocational earnings-related rehab.	1,068 (1,007-1,132)	0,03	1,080 (1,019-1,144)	0,009	1,077 (1,017-1,142)	0,01
**Employed at the beginning of the SA period**						
Any rehabilitation	1,046 (1,014-1,078)	0,004	1,050 (1,019-1,083)	0,002	1,058 (1,025-1,093)	0,001
Kela's rehabilitation	1,042 (1,005-1,080)	0,03	1,035 (0,996-1,076)	0,08	1,042 (0,999-1,087)	0,06
Vocational rehabilitation	1,141 (1,035-1,258)	0,008	1,162 (1,045-1,292)	0,006	1,201 (1,069-1,349)	0,002
Medical rehabilitation	0,936 (0,755-1,161)	0,54	0,967 (0,775-1,206)	0,76	0,998 (0,787-1,265)	0,98
Discretionary rehabilitation	1,031 (0,986-1,079)	0,17	1,027 (0,979-1,077)	0,27	1,025 (0,973-1,080)	0,34
Rehabilitative psychotherapy	1,006 (0,933-1,084)	0,87	0,976 (0,897-1,061)	0,56	1,016 (0,921-1,120)	0,75
Vocational earnings-related rehab.	1,070 (1,009-1,134)	0,03	1,076 (1,016-1,139)	0,01	1,074 (1,015-1,137)	0,02
**Not employed at the beginning of the SA period**						
Any rehabilitation	1,044 (0,993-1,098)	0,09	1,060 (1,007-1,115)	0,03	1,048 (0,996-1,103)	0,07
Kela's rehabilitation	1,047 (0,990-1,106)	0,10	1,061 (1,003-1,121)	0,04	1,043 (0,986-1,103)	0,14
Vocational rehabilitation	1,045 (0,961-1,136)	0,30	1,046 (0,960-1,140)	0,30	1,009 (0,929-1,096)	0,83
Medical rehabilitation	1,335 (1,008-1,768)	0,04	1,306 (0,986-1,730)	0,06	1,389 (1,051-1,836)	0,02
Discretionary rehabilitation	1,045 (0,958-1,141)	0,32	1,067 (0,979-1,164)	0,13	1,047 (0,958-1,145)	0,30
Rehabilitative psychotherapy	1,026 (0,910-1,157)	0,67	1,043 (0,924-1,177)	0,49	1,021 (0,899-1,158)	0,75
Vocational earnings-related rehab.	1,033 (0,866-1,232)	0,71	1,059 (0,890-1,259)	0,51	1,043 (0,874-1,244)	0,64

Table S2. Impact of the sickness allowance reform on labour market outcomes TWO YEARS after passing the 30, 60 and 90 sickness allowance (SA) days, ITS- analysis adjusted for seasonality and the demographic confounders, incidence rate ratios (IRR) and 95% confidence intervals (95% CI)

	30 SA days		60 SA days		90 SA days	
	IRR (95% CI)	p-value	IRR (95% CI)	p-value	IRR (95% CI)	p-value
**All**						
Employed	1,002 (0,991-1,013)	0,71	1,007 (0,990-1,023)	0,43	0,994 (0,972-1,017)	0,59
Unemployed	0,948 (0,921-0,976)	<0,001	0,934 (0,904-0,966)	<0,001	0,943 (0,910-0,979)	0,002
Sickness allowance	1,040 (0,989-1,094)	0,13	1,041 (0,978-1,107)	0,20	0,975 (0,913-1,041)	0,44
Disability pension	1,007 (0,980-1,034)	0,63	1,020 (0,994-1,047)	0,13	1,023 (0,998-1,048)	0,07
Other	1,011 (0,983-1,041)	0,44	1,000 (0,968-1,032)	0,99	1,029 (0,993-1,067)	0,12
**Employed at the beginning of the SA period**						
Employed	0,997 (0,988-1,007)	0,58	0,993 (0,979-1,008)	0,37	0,984 (0,964-1,005)	0,14
Unemployed	0,976 (0,935-1,018)	0,24	0,966 (0,918-1,017)	0,18	0,977 (0,922-1,035)	0,42
Sickness allowance	1,016 (0,961-1,076)	0,57	1,033 (0,962-1,108)	0,37	0,982 (0,908-1,062)	0,64
Disability pension	1,016 (0,984-1,049)	0,33	1,016 (0,985-1,049)	0,31	1,028 (0,996-1,061)	0,09
Other	1,007 (0,974-1,040)	0,69	0,990 (0,951-1,030)	0,61	1,012 (0,966-1,060)	0,60
**Not employed at the beginning of the SA period**						
Employed	1,010 (0,955-1,068)	0,73	1,053 (0,981-1,131)	0,15	1,010 (0,926-1,102)	0,81
Unemployed	0,930 (0,899-0,962)	<0,001	0,915 (0,876-0,955)	<0,001	0,926 (0,885-0,969)	0,001
Sickness allowance	1,091 (0,978-1,218)	0,11	1,065 (0,945-1,201)	0,30	0,969 (0,856-1,096)	0,61
Disability pension	1,006 (0,969-1,044)	0,77	1,024 (0,987-1,062)	0,20	1,030 (0,995-1,065)	0,09
Other	1,041 (0,997-1,087)	0,07	1,021 (0,971-1,074)	0,41	1,035 (0,978-1,096)	0,23
